# Discrimination Learning, Reversal, and Set-Shifting in First-Episode Schizophrenia: Stability Over Six Years and Specific Associations with Medication Type and Disorganization Syndrome

**DOI:** 10.1016/j.biopsych.2009.05.016

**Published:** 2009-09-15

**Authors:** Verity C. Leeson, Trevor W. Robbins, Elizabeth Matheson, Samuel B. Hutton, María A. Ron, Thomas R.E. Barnes, Eileen M. Joyce

**Affiliations:** a UCL Institute of Neurology, London, United Kingdom; b Imperial College Faculty of Medicine, London, United Kingdom; c Department of Experimental Psychology, University of Cambridge, Cambridge, United Kingdom; d University of Sussex, Brighton, United Kingdom

**Keywords:** Cognition, first-episode, IDED, reversal learning, schizophrenia, set-shifting

## Abstract

**Background:**

The intradimensional/extradimensional (IDED) task assesses different forms of learning from feedback. Limited evidence suggests that attentional set-shifting deteriorates over time in schizophrenia. We tested this hypothesis and examined the specificity of learning impairments identified by this task.

**Method:**

Two hundred sixty-two first-episode patients and 76 healthy control subjects, matched for age and premorbid IQ, were tested; 104 patients and 25 control subjects were reassessed 1 and 3 years later, and 31 patients were reassessed additionally 6 years later.

**Results:**

Patients showed impaired set-shifting that correlated with current IQ and working memory, but there were no impairments when subgroups were matched on current IQ. In contrast, patients showed marked impairments in rule reversal learning that survived correction for IQ, were present in the context of intact rule abstraction, and correlated with disorganization symptoms. Patients prescribed second-generation antipsychotics were worse on set-shifting compared with first-generation, a finding not explained by demographic data, illness characteristics, or IQ. Patients and control subjects showed stable IDED performance over the first 6 years of illness, although set-shifting was inconsistent over the first year. Those with residual negative symptoms were more likely to fail the set-shifting stage at follow-up.

**Conclusions:**

First-episode schizophrenia patients can learn and generalize rules but are inflexible when rules change, reflecting reduced responsiveness to negative feedback and difficulty in switching attention. Rule-reversal is a promising target for translational studies, because it is specific, clinically relevant, and might reflect orbitofrontal dysfunction. Set-shifting is related to poor function more generally but might be sensitive to medication effects and valuable for clinical trials.

Being able to learn by experience is crucial for effective negotiation of the environment. Patients with schizophrenia show learning impairments on episodic memory tasks (1), but less is known about how learning is shaped by positive and negative feedback (2). This is relevant, because recent models of experience-dependent learning implicate cognitive and neural substrates considered dysfunctional in schizophrenia. For example, the dopaminergic input to striatum and prefrontal cortex is thought to signal unexpected events and facilitate a shift in attention to promote new learning (3), and prefrontal attentional processes have been shown to selectively “tune” into task-relevant stimuli during learning (4).

The Cambridge Neuropsychological Test Automated Battery (CANTAB) intradimensional/extradimensional (IDED) task (5,6) lends itself to the study of learning in schizophrenia, because subjects progress through different stages in which responses can be modified by feedback. As well as discrimination learning, other types of learning important for adapting to a changing environment can be measured. These include learning to change responses when stimuli are no longer relevant (reversal learning), learning to generalize responses from a particular stimulus to others in the same category or dimension (rule abstraction), and shifting attention to a different stimulus dimension when the current dimension is no longer fruitful (attentional set-shifting). Studies of this task in nonhuman primates and man have shown that these different forms of learning are mediated by different neural processes (6). For example, a double-dissociation has been demonstrated implicating lateral prefrontal cortex in attentional set-shifting and orbitofrontal cortex in reversal learning (7–10), and dopamine innervation of prefrontal cortex seems to “stabilize” attentional-set and promote rule learning (11,12).

Previous studies of schizophrenia have found different patterns of deficit in first-episode patients compared with those with long-standing illness. Most first-episode patients pass the test, with the remainder failing at the attentional set-shifting stage (13,14). Patients with longer illness durations are more impaired, mainly in set-shifting but also at earlier stages (15–19). Some studies (20,21) but not all (17,19,22) have found an inverse relationship between illness duration and set-shifting ability, and there are preliminary reports of deterioration in performance 1 year or 7 years after the first episode (23,24). Ceaser *et al.* (19) recently found that, in contrast to schizophrenia patients, their healthy siblings were only minimally impaired on the task, suggesting that IDED performance is not an intermediate phenotype of the genetic risk for schizophrenia (19) but might be sensitive to illness processes that deteriorate over time after illness onset. Because there are few controlled longitudinal studies of specific cognitive functions after the first episode (25), a finding of deterioration in IDED performance would be of clinical importance by providing a target for future pharmacological remediation (26). In the current study, we tested this hypothesis by assessing IDED performance in schizophrenia patients at first episode and again 1 year and 3 and 6 years after onset and compared this with healthy control subjects tested at the same time points. On the basis of previous studies we predicted that set-shifting performance would deteriorate over time.

Also, of relevance is whether the IDED task identifies specific forms of learning impairment. For example, the studies of long-standing schizophrenia that have examined current IQ effects on IDED have reached different conclusions about the specificity of set-shifting (15,16,18,19), and no study has examined this at first-episode. Cognitive Neuroscience Treatment Research to Improve Cognition in Schizophrenia (CNTRICS) has recently recommended IDED as a task-measuring rule generation and selection for possible translation into clinical trials, partly on the basis of its widespread use in animal behavioral pharmacology (25). Thus, determining the specificity of any learning impairment is important, especially at first episode when remediation strategies are likely to be most effective. We therefore assessed the impact of generalized cognitive functioning on performance at the different stages of the task by measuring premorbid and current IQ and also examined the relationship between IDED performance and working memory. To test the clinical relevance of our findings, we examined performance variables in relation to symptoms and social function and compared the effect of first- and second-generation medication.

## Methods and Materials

### Participants

Patients were recruited in their first psychotic episode and had <12 weeks antipsychotic medication exposure; 262 individuals with a diagnosis of schizophrenia (*n* = 235), schizophreniform (*n* = 3), or schizoaffective disorder (*n* = 24) were included (see [27] for more details). The baseline data of 136 patients were reported previously (20). The control group comprised 76 healthy volunteers, recruited from local job centers, schools, and hospitals, without a prior history of psychiatric illness in themselves or first-degree relatives, head injury, neurological or endocrine disorder, or drug or alcohol dependence. Research Ethics Committee permission was obtained. Participants gave written informed consent and were paid an honorarium for their time. One hundred four patients and 25 control subjects were reassessed twice; mean weeks to second and third assessments: patients = 65.33 (34.77) and 177.91 (77.66), respectively; control subjects = 60.48 (33.37) and 129.75 (44.08), respectively.

### Clinical Assessments

Psychotic symptoms were assessed with the Scales for the Assessment of Positive and Negative Symptoms (28,29), and scores for the three syndromes of schizophrenia (30) were calculated. Social function was assessed with the Social Function Scale (31). Duration of untreated psychosis (DUP) was calculated as the time from onset of psychotic symptoms to first treatment with antipsychotic medication (32).

### Neuropsychological Assessment

For the IDED task, subjects learn a series of visual discriminations in which one of two stimuli is correct. Feedback from the computer indicates whether a choice is correct (green tick, high tone) or incorrect (red cross, low tone). The rule is changed after six consecutive correct choices. If the learning criterion is not achieved within 50 trials, the test is discontinued. Stage 1 (simple discrimination [SD]) requires learning of the correct stimulus from a choice of two shapes; at Stage 2 (simple reversal [SR]) the previously irrelevant shape becomes correct. At Stage 3 (compound discrimination [C_D]) a second dimension (line) is introduced alongside the shape dimension with each of the two stimuli containing a shape and line; subjects need to continue selecting the shape dimension. At Stage 4 (compound discrimination [CD]), the lines are superimposed onto shapes in each stimulus, and responding to the previous shape is required. At Stage 5 (compound reversal [CR]) the previously incorrect shape now becomes the correct response. Thus, at stages 1–5, the exemplars are the same, and subjects are required to respond to the same dimension of shape. Stage 6 (intradimensional shift [IDS]) tests the veracity of rule learning as new compound line and shape exemplars are introduced, but the same dimension (shape) remains correct. Selection of the previously incorrect shape pattern is required at Stage 7 (intradimensional reversal [IDR]). Stage 8 tests attentional set-shifting (extradimensional shift [EDS]) as the previously irrelevant dimension (line) now becomes relevant, and one of the line patterns becomes the correct response. At Stage 9 (extradimensional reversal [EDR]) the previously incorrect line becomes the required response. The various stages of this task require simple discrimination learning, compound discrimination learning, abstraction, attentional set-shifting, and reversal learning.

Other CANTAB executive tasks were employed for comparison. Working memory manipulation was measured with the Spatial Working Memory task, whereby participants need to recall where previous “tokens” were found from a random array of “boxes” to maximize success at finding subsequent “tokens”. The number of search errors was measured. Working memory span was measured with a version of the Corsi block test, requiring the recall of consecutively presented boxes at different spatial locations. Premorbid IQ was estimated with the National Adult Reading Test (NART-R) (33). Current IQ was calculated from four subtests of the Wechsler Adult Intelligence Scale-Revised (34) or Wechsler Adult Intelligence Scale-III (35), which have been shown to provide reliable measures of full-scale IQ in psychosis (36,37).

### Analyses

Univariate analysis of variance (ANOVA) was used for demographic data and linear neuropsychological measures. Task analyses included only patients attempting a given stage; numbers passing were analyzed with *χ*^2^ tests and pass rates for each stage with likelihood ratio analysis (38). Errors were not normally distributed and could not be normalized by mathematical transformation and thus were compared with Kruskal–Wallis comparisons. For repeated measures, errors were analyzed with Friedman's ANOVA and pass rates with Cochran's Q. Correlations among errors, symptoms, and social function were analyzed with Spearman's ρ. Logistic regression was employed to determine measures that predicted passing the test.

## Results

### Demographic Data

There were no statistically significant differences between the baseline groups on age or premorbid IQ (Table 1); there were more women in the control group. Followed-up subjects did not differ significantly on any variable.

### Baseline Performance

#### Pass/Fail

**Figure 1:**
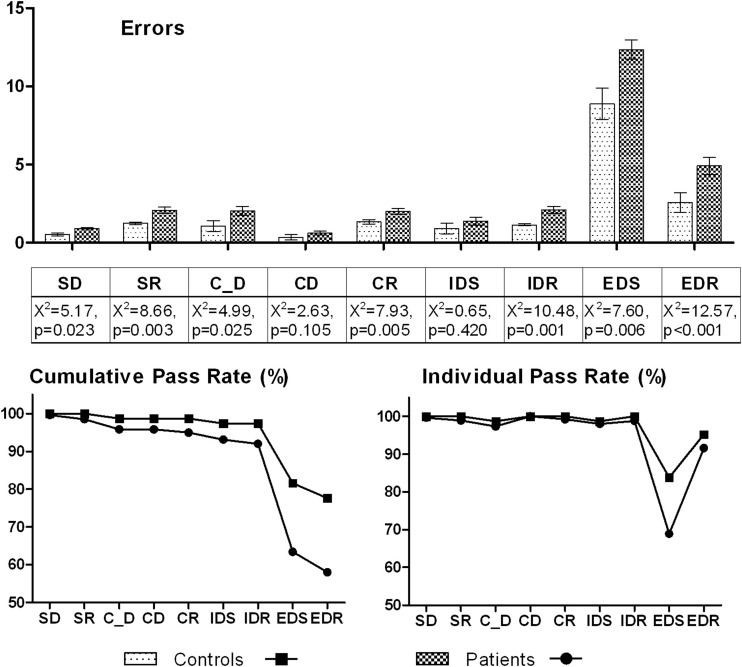
Baseline attentional set shifting task performance. Upper panel: for patients and control subjects, mean errors with standard error bars are shown on the y axis for each stage denoted along the x axis. For each stage, Kruskal–Wallis comparisons are given. Lower panel: on the left the cumulative pass rate (%) is given on the y axis for each stage denoted along the x axis. For each stage this refers to the total number of patients and control subjects who have passed up until and including the current stage. On the right, the pass rate for the individual stage (%) is given on the x axis for each stage denoted on the y axis. This refers to the number of patients and control subjects who actually attempted that particular stage, having passed the previous stage. SD, simple discrimination; SR, simple reversal; C_D, compound discrimination; CD, compound discrimination; CR, compound reversal; IDS, intradimensional shift; IDR, intradimensional reversal; EDS, extradimensional shift; EDR, extradimensional reversal.

More control subjects than patients completed all nine stages (*χ*^2^ = 9.32, *p* = .002) (Figure 1). Significantly more patients failed at the EDS stage (2i = 6.80, *p* = .009) but not at any other stage (all *p* values > .1). Patients made significantly more errors on the first two learning stages, SD and C_D, but not at later CD and IDS stages; they also made more errors at the EDS stage. Patients made significantly more errors at every reversal stage; even those patients passing EDS subsequently made more errors at EDR.

#### Correlations

When we examined only those patients reaching the EDS stage (*n* = 242), errors at EDS did not correlate with errors at the SD (ρ = −.01, *p* = .881) or C_D (ρ = −.06, *p* = .345) stages or the summed errors from reversal stages before EDS (ρ = −.01, *p* = .957). The EDS errors correlated with current IQ (*r* = −.40, *p* < .001), working memory span (*r* = −.23, *p* < .001), and working memory manipulation (*r* = .23, *p* < .001), so that better ability was related to fewer errors.

#### IQ Effects

Current IQ was lower in patients (mean [standard deviation] IQ, patients [*n* = 236]: 88.17 [16.54]; control subjects: 100.93 [12.69]) [*F*(1,311) = 38.04, *p* < .001]. We therefore examined matched groups with a current IQ in the average range or above (IQ ≥ 90; patients *n* = 98, mean 103.82 [11.58]; control subjects *n* = 63, mean 104.60 [10.52]) [*F*(1,160) = −.19, *p* = .663]. The overall pass rate in this subset was higher for both groups (patients: 73.5% vs. 58.4%; control subjects: 84.1% vs. 77.6%), which was not different (*χ*^2^ = 2.51, *p* = .113). Both groups passed SD, SR, CD, and CR; and there were no pass/fail differences at other stages (all *p* > .1) including EDS (2i = 1.44, *p* = .231). Increased reversal errors in patients were evident at CR (*χ*^2^ = 5.47, *p* = .017), IDR (*χ*^2^ = 5.07, *p* = .024), and EDR (*χ*^2^ = 3.31, *p* = .069).

#### Clinical Relationships

The strongest finding was that reversal errors correlated significantly with disorganization syndrome at every stage, particularly with respect to positive formal thought disorder (e.g., SR stage: ρ = .21, *p* < .001) (Table 2). There were no differences between those passing or failing EDS on social function [*F*(1,223) = 2.75, *p* = .100] or negative [*F*(1,240) = 1.52, *p* = .219], positive [*F*(1,240) = .01, *p* = .981], and disorganization [*F*(1,240) = .49, *p* = .486] syndromes.

#### Medication Effects

Fourteen were drug-free, 61 were prescribed first-generation antipsychotics (haloperidol *n* = 15; sulpiride *n* = 23; droperidol *n* = 9; trifluoperazine *n* = 10; thioridazine *n* = 2; chlorpromazine *n* = 1; flupentixol *n* = 1), and 172 were prescribed second-generation antipsychotics (risperidone *n* = 55; olanzapine *n* = 102; amisulpride *n* = 8; quetiapine *n* = 3; clozapine *n* = 1; aripiprazole *n* = 3); 15 were taking a combination and excluded from the analyses. Sixty-four percent of the drug-free group, 74% of the first-generation antipsychotic group, and 52% of the second-generation antipsychotic group passed all stages of the test, and this difference was significant (*χ*^2^ = 8.74, *p* = .013). Pairwise analyses revealed that more patients prescribed first-generation passed the task compared with those prescribed second-generation antipsychotics (*χ*^2^ = 8.50, *p* = .004). Stage-by-stage analysis showed that only passing at EDS stage was different between groups (*χ*^2^ = 9.12, *p* = .010; all other stages *p* > .1). There were no differences between those taking first- and second-generation antipsychotics on any clinical or cognitive variable that might mediate this difference (Supplement 1). There were no differences between groups prescribed anticholinergics (*n* = 52) or not (*n* = 210) on passing the task (*χ*^2^ = .55, *p* = .457).

### Change Over 1 Year and 3 Years

#### Pass/Fail

**Figure 2:**
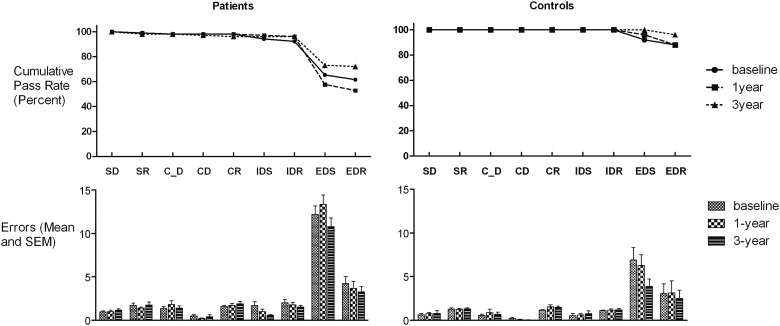
The upper panel shows cumulative pass rates for patients and control subjects at baseline, 1 year follow-up, and 3-year follow-up for those subjects who completed all three assessments. This denotes the percentage of subjects passing up to and including the current stage. The lower panel shows the number of errors committed at each stage at the three time points for patients and control subjects. Abbreviations as in Figure 1.

The number of patients passing the entire task changed over time (baseline 63.5%, 1-year 52.9%, 3-year 72.1%; *Q* = 10.75, *p* = .005), reflecting a trend for deterioration from baseline to 1 year (*Q* = 3.67, *p* = .056), but no difference between baseline and 3 years (*Q* = 1.98, *p* = .160) (Figure 2, Table 3). The control subjects did not show significant change over time (baseline 88%, 1 year 88%, 3-year 96%; *Q* = 2.67, *p* = .264).

The patients showed a significant change over time in passing EDS (baseline: 70.8%, 1-year: 59.6%, 3-year: 75.3%; *Q* = 6.78, *p* = .034), reflecting significant decline from baseline to 1-year (*Q* = 4.48, *p* = .034), but no difference between baseline and three years (*Q* = 1.60, *p* = .206). The control subjects did not change over time (baseline: 92%, 1-year: 96%, 3-year: 100%; *Q* = 2.00, *p* = .368). There was no significant change in pass rate at other stages (patients: all *p* > .1 except IDS: *Q* = 5.20, *p* = .074; control subjects: all stages passed except EDR: *Q* = 3.00, *p* = .223).

Passing the test at the first assessment predicted passing at the second assessment in patients (*χ*^2^ = 14.08, *p* < .001, Wald = 12.91, exp[β] = .204) and control subjects (*χ*^2^ = 6.39, *p* = .011, Wald = 5.48, exp[β] = .024).

#### Errors

Friedman's ANOVA showed that the patients did not change significantly in the number of errors made at any stage (all *p* > .1).

#### Clinical Relationships

When patients passing and failing EDS at the second assessment were compared on concurrent clinical features, there were no differences in social function [*F*(1,98) = .13, *p* = .716] and the presence of positive (*χ*^2^ = .71, *p* = .400) or disorganization (*χ*^2^ = .38, *p* = .536) symptoms. Significantly more patients failing EDS had residual negative symptoms (*χ*^2^ = 6.91, *p* = .001). This pattern was similar at the third assessment [social function: *F*(1,99) = .01, *p* = .989; negative syndrome: *χ*^2^ = 4.88, *p* = .027; positive syndrome: *χ*^2^ = .63, *p* = .428; disorganization syndrome: *χ*^2^ = 1.35, *p* = .245].

### Six-Year Follow-Up

**Figure 3:**
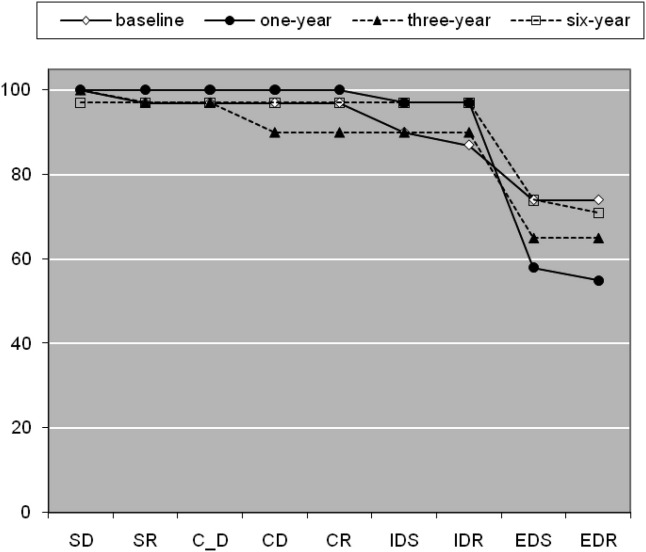
Cumulative pass rates by stage at baseline, 1 year, 3-year, and 6-year follow-up assessments in the subset of patients who had completed all four assessments (*n* = 31). This denotes the % subjects passing up to and including the current stage. Abbreviations as in Figure 1.

Thirty-one patients received a further assessment a mean of 322.26 (91.08) weeks after their first assessment (Figure 3). They had a mean age of 25.10 (7.93) years at baseline and mean NART IQ of 99.87 (12.21), comprised 25 men and 6 women, and did not differ from remainder of patients in the larger followed-up group [premorbid IQ: *F*(1,103) = .22, *p* = .637; age: *F*(1,103) = .57, *p* = .452; gender: *χ*^2^ = 2.71, *p* = .100]. There was a trend for a change over time in overall pass rate (*Q* = 6.58, *p* = .087). Post hoc analysis showed that more patients failed at 1 year than baseline (*Q* = 6.40, *p* = .01), but there were no differences between baseline and three years (*Q* = 2.57, *p* = .11) or baseline and 6 years (*Q* = .50, *p* = .48). There were no differences in pass rate at EDS (*Q* = 3.78, *p* = .286) or any other stage (all *p* > .1) over time or in the number of errors made at any stage over time except SR (*χ*^2^ = 8.08, *p* = .044; all other stages *p* > .1).

## Discussion

In this study of first-episode schizophrenia, we used a single task to examine cognitive processes essential for successful adaptation to a changing environment. These were discrimination learning and reversal, rule abstraction, and set-shifting (5,6). There were several major findings in addition to confirming the well-established impairment in set-shifting (39,40). First, schizophrenia patients showed a marked impairment in reversal learning that was present in the context of intact rule abstraction and was linked to disorganization symptoms. Second, set shifting was poorer in those patients prescribed second-generation than first-generation antipsychotic medication. Third, performance seemed stable when tested over a period of 6 years from illness onset.

Patients showed the most pronounced deficit at the attentional set-shifting stage (EDS) of the task, when they were required to inhibit their current responding to a stimulus dimension and learn to respond to a different dimension. This is also the most common finding in previous studies of first-episode or long-standing schizophrenia (14–16,18,19,40). Performance of EDS entails several cognitive processes including shifting the focus of attention and learning from feedback. In a positron emission tomography study, Rogers *et al.* (8) found that the dorsolateral prefrontal cortex (DLPFC) was more active during EDS than during performance at the rule abstraction stage (IDS). Hampshire and Owen (9) examined EDS with a functional magnetic resonance imaging task in which the subjects repeatedly shifted attention between stimulus dimensions, thus disambiguating the attentional shift from learning how to solve the problem. They found that ventrolateral prefrontal cortex (VLPFC) was specifically active during the attentional shift, whereas DLPFC activity reflected more the strategic and working memory demands involved in the EDS problem solution. This finding informs the interpretation of our findings, because EDS errors correlated significantly with current IQ and independent measures of working memory but not with within-task measures of discrimination learning and reversal. Furthermore, when we examined subgroups of patients and control subjects matched for current IQ, there were no EDS pass/fail differences. This suggests that the EDS impairment in schizophrenia reflects the general problem-solving requirements of this stage, a conclusion supported by a large-scale study of long-standing schizophrenia showing pronounced IQ effects on EDS performance (20) and compatible with the well-established DLPFC deficits in this patient group (41). Our finding that failing EDS, when retested at 1 year and 3 years, was related to the presence of enduring negative symptoms also supports the contention that EDS is sensitive to generally poor function in patients with schizophrenia.

In contrast, the patients had striking difficulty with reversal learning, which survived correction for current IQ. They made more errors than control subjects at every reversal stage at baseline, and this pattern persisted when tested on two subsequent occasions over 3 years. Our findings are compatible with a study (39) that used a task analogous to the SD and SR stages in the attentional set-shifting task but with probabilistic contingencies; patients acquired the discrimination but were impaired on reversal. In addition, Murray *et al.* (14) found that those patients who attempted all stages of the attentional set-shifting task made more total reversal errors than control subjects. When we compared subgroups matched on current IQ, reversal learning was still impaired, with patients making more errors on three of the four reversal stages. Thus, regardless of current intellectual function, patients were less able than control subjects to modify their behavior in response to negative feedback.

This finding is evidence for dysfunction of orbitofrontal cortex (OFC). Studies have shown that the OFC is involved in the control of responding in the face of changing reward/punishment contingencies (42,43). The OFC is activated in man and nonhuman primates during reversal learning (44–46), and reduced OFC activity is associated with poor reversal learning (47), supporting the view that this area is active when negative feedback signals the need to change a response set. Hampshire and Owen (9) found that lateral OFC was specifically active during an attentional shift after negative feedback rather than during the negative feedback itself and suggest this area is responsible for implementing a response shift rather than processing negative feedback. Compatible with this are the findings from other studies that suggest that OFC serves to maintain the representation of the negative value of stimuli for action selection (48–50). In our study, the persistent reversal learning deficit might be secondary to the diminished ability of patients to represent the value of negative feedback. An alternative explanation is that patients learned to ignore the alternate aspects of the stimulus array. However, Elliott *et al.* (15) found that patients with schizophrenia were impaired on this task, not because of learned irrelevance but because they perseverated on a previously reinforced dimension, a finding compatible with an explanation of reduced responsivity to negative feedback. A relevant study (51) showed that patients with schizophrenia had difficulty in “translating experience into action.” Thus, compared with control subjects, patients described similar depths of emotion to positive, negative, and neutral stimuli but acted as if the stimuli were less emotionally arousing, and this was particularly evident for stimuli with negative valence. This might also explain our previous findings with a gambling task sensitive to OFC function (42). Patients with first-episode schizophrenia were able to judge correctly the magnitude of rewards/punishments that varied over trials but were less able than control subjects to act on this information by adjusting how many points they “bet” on any one trial (52). Similarly, Heerey *et al.* (53) found that patients with schizophrenia made suboptimal decisions, especially concerning potential losses on their gambling task.

We found a significant correlation between reversal errors at every stage and the disorganization syndrome, particularly the formal thought disorder (FTD) component. Although the size of the correlations was small, other findings suggest that this link might be important. A meta-analysis found that, of all cognitive impairments associated with FTD, one of the strongest was executive inhibition (54). Our findings shed light on the specificity of this association, because disorganization did not correlate with other aspects of IDED performance. Thus, FTD might reflect a difficulty in inhibiting a previous response or thinking pattern rather than an inability to shift attention and respond appropriately. It is also noteworthy that a neuroimaging study of schizophrenia found that reduced volume of OFC was associated with increased FTD (55). Tentatively, these findings suggest that reversal deficits in schizophrenia might be a clinically promising target for future remediation, because they are linked not only to a specific neural process but also to a clinical syndrome.

The patients also made more errors at the first two discrimination learning stages (SD and C_D) but not at later learning stages involving compound discrimination and rule abstraction. Thus, they had initial difficulty in forming an attentional set, but once acquired, they were able to apply successfully the rule governing responding to other situations even though the stimuli were more complex or changed in their physical attributes. This pattern of responding was the same when retested 1 year and 3 years later. Previous studies of schizophrenia have suggested that this instability of initial learning reflects dopamine dysregulation (14,18). A study in the marmoset has shown that prefrontal dopamine depletion impairs the ability to acquire an attentional set particularly at the rule abstraction stage (IDS) (12). Because patients were medicated in these studies and medication, if anything, tends to improve executive function, it is possible that intact rule abstraction represented a positive medication effect on learning.

Those taking second-generation antipsychotics, compared with first-generation antipsychotics, failed the task more frequently due to impaired EDS performance. Because we could not identify any other differences between the groups that might mediate this finding, it suggests that more selective dopamine D2 receptor blockers had a beneficial effect on set-shifting, possibly by stabilizing dopamine activity in frontostriatal circuitry. In the absence of a randomized clinical trial, these results must be regarded tentatively, especially because we could not establish whether this effect persisted because most patients taking first-generation antipsychotics had been switched to second-generation drugs by 1 year follow-up. Furthermore, we did not find this effect on working memory, another executive function that is compromised in schizophrenia and thought to be modulated by forebrain dopamine.

Our prediction of deterioration at the EDS stage was not borne out when a large group of patients were retested 3 years after onset. To examine whether our follow-up period might not have been sufficiently long, we examined a smaller group tested again at 6 years and found no deterioration. Therefore, we were unable to replicate a preliminary report of worse performance over a similar follow-up period and in a similar number of patients (24). However, we did confirm our own preliminary finding (23) that fewer patients passed the EDS stage when tested at 1 year. Thus, a proportion of patients who successfully shifted set at illness onset failed to do so 1 year later, suggesting that this cognitive function is unstable in the early stages of the illness. It is possible that long-term treatment stabilized executive control over the following years. Alternatively, ceiling effects in control subjects might have masked a widening gap between patient and control performance whereby practice counteracted deteriorating functioning in patients but could not increase success in control subjects. Against this is that the reduced EDS pass rate at 1 year was not mirrored by changes in error rates at any stage over three testing sessions.

Taken together our findings suggest that patients with first-episode schizophrenia can learn and generalize rules but are inflexible when rules change, reflecting both reduced responsiveness to negative feedback and difficulty in switching attention. This profile seems to represent a stable trait of the illness, with the proviso that set-shifting seems to fluctuate in the first year. Given that EDS is also somewhat dependent on working memory and general problem solving, our findings suggest that attentional set-shifting per se might not be a particularly illuminating construct in schizophrenia. The more rigid and inflexible behavior in schizophrenia might be better captured by reversal learning, because this measure was found to be relatively independent of IQ and associated with a specific clinical syndrome at illness onset and has distinct neural substrates. Nevertheless, the finding that EDS was sensitive to medication effects suggests that it might be a valuable measure in the assessment of pharmacological remediation of cognition in randomized controlled clinical trials.

## Figures and Tables

**Table 1:** Demographic Data for Patients and Control Subjects Completing One Neuropsychological Assessment and the Subgroups Completing all Three Assessments

	Initial Assessment			Three Assessments		
	Patients	Control Subjects	Comparison	Patients	Control Subjects	Comparison
*N*	262	76		104	25	
Gender	189 M/73 F	40 M/36 F	*χ*^2^ = 10.26, *p* = .001	72 M/32 F	13 M/12 F	*χ*^2^ = 2.66, *p* = .103
Age in Yrs	25.74 (7.90)	26.42 (7.63)	*F*(1,337) = .44, *p* = .506	26.02 (8.09)	26.92 (7.66)	*F*(1,128) = .26, *p* = .615
Premorbid IQ (NART)	95.78 (13.44)	98.74 (10.76)	*F*(1,323) = 3.08, *p* = .080	98.98 (12.50)	97.83 (11.24)	*F*(1,218) = 1.4, *p* = .113

National Adult Reading Test (NART) not completed in 14 patients.

**Table 2:** Spearman's Correlations Between Errors at Each Stage and Clinical Measures

Stage	SD	SR	C_D	CD	CR	IDS	IDR	EDS	EDR
Negative Syndrome	.12	.15^a^	.06	.08	.01	−.01	.06	.06	.06
Positive Syndrome	.08	.12	.01	.08	.08	−.03	.02	.09	.12
Disorganization Syndrome	.03	.21^c^	.13^a^	.00	.14^a^	−.06	.14^a^	−.04	.15^a^
Social Function	−.05	−.10	−.17^b^	−.07	−.15^a^	.00	−.11	−.02	.01

SD, simple discrimination; SR, simple reversal; C_D, compound discrimination; CD, compound discrimination; CR, compound reversal; IDS, intradimensional shift; IDR, intradimensional reversal; EDS, extradimensional shift; EDR, extradimensional reversal.

a *p* < .05.

b *p* < .01.

c *p* < .001.

**Table 3:** Errors (Standard Deviation) at Each Stage over the Three Assessments in Patients and Control Subjects that Completed all Three Assessments with Separate Repeated Measures Analyses (Friedman's ANOVA) for Patients and Control Subjects

	Patients				Control Subjects			
	Baseline	1 Yr	Three-Yr	Comparison	Baseline	1 Yr	Three-Yr	Comparison
SD	.95 (1.19)	1.01 (1.62)	1.15 (1.68)	*χ*^2^ = 3.02, *p* = .221	.56 (.65)	.68 (.80)	.72 (1.49)	*χ*^2^ = .84, *p* = .656
SR	1.72 (2.56)	1.40 (1.12)	1.77 (3.34)	*χ*^2^ = 1.64, *p* = .439	1.20 (.65)	1.24 (.52)	1.32 (.63)	*χ*^2^ = 1.65, *p* = .439
C_D	1.58 (3.32)	1.82 (4.45)	1.88 (4.15)	*χ*^2^ = .34, *p* = .843	.48 (.71)	.84 (2.01)	.56 (1.26)	*χ*^2^ = .78, *p* = .679
CD	.75 (2.74)	.69 (3.46)	.88 (4.10)	*χ*^2^ = 1.68, *p* = .432	.20 (.58)	.08 (.28)	.04 (.20)	*χ*^2^ = 1.20, *p* = .549
CR	1.81 (2.60)	2.16 (3.82)	2.57 (4.68)	*χ*^2^ = .91, *p* = .634	1.12 (.33)	1.48 (1.05)	1.52 (.77)	*χ*^2^ = 5.88, *p* = .053
IDS	1.93 (5.14)	1.45 (4.46)	1.53 (4.78)	*χ*^2^ = .77, *p* = .682	.52 (1.29)	.60 (.76)	.76 (1.50)	*χ*^2^ = 4.98, *p* = .083
IDR	2.90 (5.72)	2.44 (5.06)	2.44 (5.08)	*χ*^2^ = 1.16, *p* = .561	1.08 (.28)	1.16 (.80)	1.20 (.65)	*χ*^2^ = .50, *p* = .779
EDS	13.00 (9.93)	13.82 (10.87)	11.42 (10.02)	*χ*^2^ = 1.78, *p* = .410	6.80 (7.20)	6.12 (6.15)	3.80 (4.15)	*χ*^2^ = 5.28, *p* = .071
EDR	11.05 (11.21)	12.48 (11.69)	9.13 (10.69)	*χ*^2^ = 3.02, *p* = .221	4.52 (7.97)	4.00 (7.93)	2.32 (4.76)	*χ*^2^ = 1.22, *p* = .544

ANOVA, analysis of variance; other abbreviations as in Table 2.
