# Supplementary material for: Discrimination Learning, Reversal, and Set-Shifting in First-Episode Schizophrenia: Stability Over Six Years and Specific Associations with Medication Type and Disorganization Syndrome
Source: Biol Psychiatry. 2009 Sep 15;66(6):586–93. doi: 10.1016/j.biopsych.2009.05.016 (PMC2734076; doi:10.1016/j.biopsych.2009.05.016)
Supplement: Supplement 1 [file mmc1.pdf]

Supplemental Table 1. Comparison of clinical and cognitive measures in those patients prescribed first- and second-generation antipsychotic medication.

Measure	Count or Mean (Standard deviation)		Comparison
	First-generation antipsychotic	Second-generation antipsychotic	
Age at testing	26.21 (8.73)	25.42 (7.47)	F[1,232]=0.46, p=0.499
Duration of untreated psychosis (weeks)	50.21 (91.51)	43.53 (79.87)	F[1,230]=0.29, p=0.592
Negative syndrome	0.36 (0.25)	0.39 (0.27)	F[1,232]=0.55, p=0.460
Positive syndrome	0.72 (0.25)	0.70 (0.24)	F[1,232]=0.31, p=0.580
Disorganisation syndrome	0.40 (0.28)	0.43 (0.31)	F[1,232]=0.67, p=0.416
Social function	110.10 (10.79)	110.78 (11.04)	F[1,232]=0.14, p=0.706
Premorbid IQ	97.53 (14.91)	95.02 (13.11)	F[1,220]=1.36, p=0.247
Current IQ	91.08 (16.60)	86.78 (16.28)	F[1,226]=2.69, p=0.102
Working memory span	5.45 (1.38)	5.40 (1.45)	F[1,230]=0.05, p=0.829
Working memory manipulation	32.82 (21.51)	34.68 (18.37)	F[1,232]=0.41, p=0.521
